# Prior Elicitation for Use in Clinical Trial Design and Analysis: A Literature Review

**DOI:** 10.3390/ijerph18041833

**Published:** 2021-02-13

**Authors:** Danila Azzolina, Paola Berchialla, Dario Gregori, Ileana Baldi

**Affiliations:** 1 Unit of Biostatistics, Epidemiology and Public Health, Department of Cardiac Thoracic Vascular Sciences and Public Health, University of Padova, 35128 Padova, Italy; danila.azzolina@uniupo.it (D.A.); dario.gregori@unipd.it (D.G.); 2 Department of Traslational Medicine, University of Eastern Piedmont, 28100 Novara, Italy; 3 Department of Clinical and Biological Science, University of Turin, 10124 Turin, Italy; paola.berchialla@unito.it

**Keywords:** prior elicitation, latent dirichlet allocation, clinical trial

## Abstract

Bayesian inference is increasingly popular in clinical trial design and analysis. The subjective knowledge derived from an expert elicitation procedure may be useful to define a prior probability distribution when no or limited data is available. This work aims to investigate the state-of-the-art Bayesian prior elicitation methods with a focus on clinical trial research. A literature search on the Current Index to Statistics (CIS), PubMed, and Web of Science (WOS) databases, considering “prior elicitation” as a search string, was run on 1 November 2020. Summary statistics and trend of publications over time were reported. Finally, a Latent Dirichlet Allocation (LDA) model was developed to recognise latent topics in the pertinent papers retrieved. A total of 460 documents pertinent to the Bayesian prior elicitation were identified. Of these, 213 (45.4%) were published in the “Probability and Statistics” area. A total of 42 articles pertain to clinical trial and the majority of them (81%) reports parametric techniques as elicitation method. The last decade has seen an increased interest in prior elicitation and the gap between theory and application getting narrower and narrower. Given the promising flexibility of non-parametric approaches to the experts’ elicitation, more efforts are needed to ensure their diffusion also in applied settings.

## 1. Introduction

The frequentist inference paradigm has been the main statistical approach to the design and analysis of clinical trials since the 1940s [1]. 

However, the improvements in statistical computing methods and the introduction of the Markov Chain Monte Carlo (MCMC) algorithm have facilitated the spread of the Bayesian methods, also in the field of clinical trials [2].

The prior distribution is a key element of Bayesian inference and represents the information about a parameter of interest that is combined with the likelihood to yield the posterior distribution. The prior information may be derived from either expert beliefs (subjective prior) or relevant empirical data (objective prior) [3,4]. 

Especially when few data are available to estimate the likelihood, for example in clinical trials in rare diseases [5] and poor accrual setting [6], an informative inference complemented with an expert elicitation procedure may be useful to translate into prior probability distribution the available expert knowledge about treatment effect [7,8]. The Bayesian priors obtained through the elicitation of expert opinion can be used to augment scarce data about treatment effect, especially in clinical trial design and analysis [9]. Eliciting expert’s opinions, in the Bayesian paradigm, may demonstrate the presence of uncertainty in treatment effect belief in a quantifiable and illustrative manner. Moreover, this information can be used to plan a study, for example, the sample size calculations [10] and interim analysis [11]. Elicited prior distributions can be used to augment the information given by scarce therapeutic data [8].

Moreover, it is interesting to consider that, the development of user-friendly interfaces, as SHELF (SHeffield ELicitation Framework) [12] or MATCH (Multidisciplinary Assessment of Technology for Healthcare) [13] software, for prior elicitation, facilitates the application of the method in the clinical research and other applied settings.

The SHELF software carries out elicitation of probability distributions for uncertain quantities from a group of experts. Each expert provides a small number of probability opinions corresponding to points on a cumulative distribution function. The SHELF tool fits a range of parametric distributions displaying them in the form of fitted probabilities and percentiles. For multiple experts, a weighted linear pool of the subjective distributions can be calculated [12]. 

Another useful tool provided in the literature is MATCH, which provides a web-based interface for the SHELF routine with the aim of being more user-friendly, including also features for the conduction of the elicitation process remotely [13].

The elicitation process is usually performed by asking the experts to report a few summaries of treatment effect, generally medians, modes, and percentiles of the probability distribution. 

Some authors assessed that the role of a facilitator is fundamental in the elicitation process. The facilitator translates some percentiles, defined by experts, into a probability distribution. This process is generally based on parametric distributions (Gamma or Beta, Student, Normal or Log-Normal) [14]. This task becomes more complicated when the opinions are asked of several experts. In this case, each expert opinion may be separately translated into distributions, and finally, it is possible to pool them into a unique prior distribution. 

The elicitation approach accounts for the subjective expert’s uncertainty about the treatment effect under investigation, and the consequences of this uncertainty in final inference can be investigated using sensitivity analysis techniques [8]. 

Quantiles information about expert beliefs is generally easier to elicit than moments [15]. Probability distributions are in several cases defined by moments, and some authors have investigated procedures to derive the parameters of a distribution using mean and standard deviation [16]. However, instead of considering direct estimates of the mean and standard deviation, it is possible to ask an expert for a specific discrete set of points on the distribution for example quantiles [17]. The mean and standard deviations can be derived from applying specific weights to the quantiles [18], or fitting distributions on the discrete points [19]. 

Quantiles information are widely adopted, to fit prior probability distributions, not only in a parametric but also in the semiparametric and non-parametric setting; for example, it is possible to ask the expert the quantiles (usually at least two) of the subjective prior distribution. These points may be plotted, and it is possible to smooth a distribution function drawn through them using a semiparametric or non-parametric representation of the expert’s opinion [20,21].

In a parametric setting, the elicitation process assumes that experts’ opinion may be represented by a good note family of probability distributions identified by hyper-parameters. Thus, the elicitation consists of the definition of appropriate values for hyper-parameters to represent the experts’ belief [7].

It is widely assessed that the main limit of a parametric approach is to constrain expert belief into a pre-specified distribution [22]. Therefore, non-parametric and semi-parametric hybrid approaches have also been proposed in the elicitation process [21].

This work is aimed to investigate the state-of-the-art of Bayesian prior elicitation methods, focusing on the discrepancy between the available methodological approaches in the statistical literature and the elicitation procedures applied within the clinical trial research. 

In this general framework, another issue is the identification of the main research topics and the definition of the peculiarities of papers using parametric and non-parametric approaches in a clinical trial concerning identified research themes. A tool that automatically allows classifying the overall elicitation literature could reduce the manual text classification burden and characterise topic patterns over the time.

## 2. Materials and Methods

### 2.1. Search Strategy

A search on the Current Index to Statistics (CIS), PubMed, Scopus, Embase, and Web of Science (WOS) electronic databases, finalised to identify all papers dealing with prior elicitation and published from 1 January 1980 to 1 November 2020, was performed. The search string “prior elicitation” was used. This search string is very general to ensure that all relevant results would be included in the final analysis. The pertinent articles were identified after duplicate removal. The overall prior elicitation literature and the articles pertaining to the prior elicitation in the clinical trial have been screened by reading the title and abstract. 

### 2.2. Overall Data Description

Summary statistics were reported to describe the corpus of papers pertinent to the prior elicitation theme. 

The prior elicitation-relevant articles have been classified in those published in “Probability and Statistics” journals (here in after referred to as Statistical papers) according to the Journal Citation Reports^®^ [23] classification. The prior elicitation publication trend according to the statistical papers versus other journals has been reported. 

As for articles concerning drug clinical trials, the frequencies of published papers have been reported according to publication time and prior elicitation methods, in parametric and in not (or semi) parametric settings. 

### 2.3. State-of-the-Art of Prior Elicitation in Clinical Trials

Methodological approaches to the prior elicitation currently used in clinical trial literature have been described, evaluating the main characteristics of parametric and non-parametric approaches adopted in trial design and analysis distinguishing by type of outcome considered in the study.

For a general comparison purpose, available methods for expert elicitation in the overall pertinent prior elicitation literature have been also reported and described. 

### 2.4. Text Mining Analysis

#### 2.4.1. LDA Algorithm

A text mining (topic model) analysis has been conducted to automatically identify the main topics characterising the overall publications on prior elicitation. The literature on clinical trials could constitute a limited subset of the total literature on prior Elicitation. For this reason, this subset was used as a validation set by classifying the documents manually and comparing the outcome of the manual classification with the automatic one. Topic modelling is an unsupervised machine learning technique that is capable of automatically clustering word groups (topics) and similar expressions that best characterise a set of documents [24]. 

#### 2.4.2. Data Pre-Processing

The titles and abstracts of prior elicitation pertinent papers have been pre-processed. Punctuation, stop words, white spaces, and numbers were removed. Redundant words (prior, elicitation, expert, Bayesian, analysis) were also removed. All words were converted to lowercase.

Once the text corpus has been cleaned, a Document-Term Matrix (DTM) has been created. A DTM is a matrix, reporting documents (articles) by rows and words by columns; a generic element of DTM is the word counts. 

To detect topics, a Latent Dirichlet Allocation (LDA) [24] analysis has been performed on the DTM matrix of pertinent articles. LDA is a technique leading to the automatic discovery of themes in a collection of documents. The method assumes that each document (articles) is a mixture of topics. Documents and words are observed elements instead topics are latent structures discovered by the LDA algorithm.

The method aims to infer the latent topic structure given the words and document. LDA recreates the documents in the corpus by adjusting the relative importance of topics in documents iteratively using a Gibbs sampler algorithm [25]. 

Gibbs sampling works by performing a random walk. The starting point of the walk is chosen at random; for this reason, it may be useful to discard the first steps (burn-in period). Overall, 10,000 iterations have been considered in the computation, and 100 draws have been discarded as burn-in. A total of five Markov chains with different starting points were generated. 

#### 2.4.3. Number of Topics

The number of topics has been chosen following the maximisation criterion of the Deveaud measure [26]. The method is based on the idea of computing distances between pairs of topics over several instances of the model while varying the number of topics. The model iterations are done by varying the number of topics of the LDA model, then estimating again the Dirichlet distributions. The optimal amount of topics is reached when the overall Kullback–Leibler dissimilarity between topics achieves its maximum value [27].

#### 2.4.4. Validation and Convergence Assessment

The algorithm has been validated on the clinical trial pertinent articles. Furthermore, the overall accuracy has been calculated, comparing the manual and automatic classification.

The convergence of the LDA algorithm has been evaluated showing the Log-Likelihood in correspondence of the first 500 iterations. If the Log-Likelihood estimate stabilises after the first iterations, then the convergence is deemed acceptable [25].

#### 2.4.5. Results

Once the algorithm has been validated, the distribution of the publication topics identified by the algorithm on the prior elicitation literature has been characterised according to the year of publication and the field of application (trial versus other pertinent literature).

Computations have been performed using R 3.3.2 [28] System with topicmodels [29] package.

## 3. Results

### 3.1. Overall Data Description

**Figure 1:**
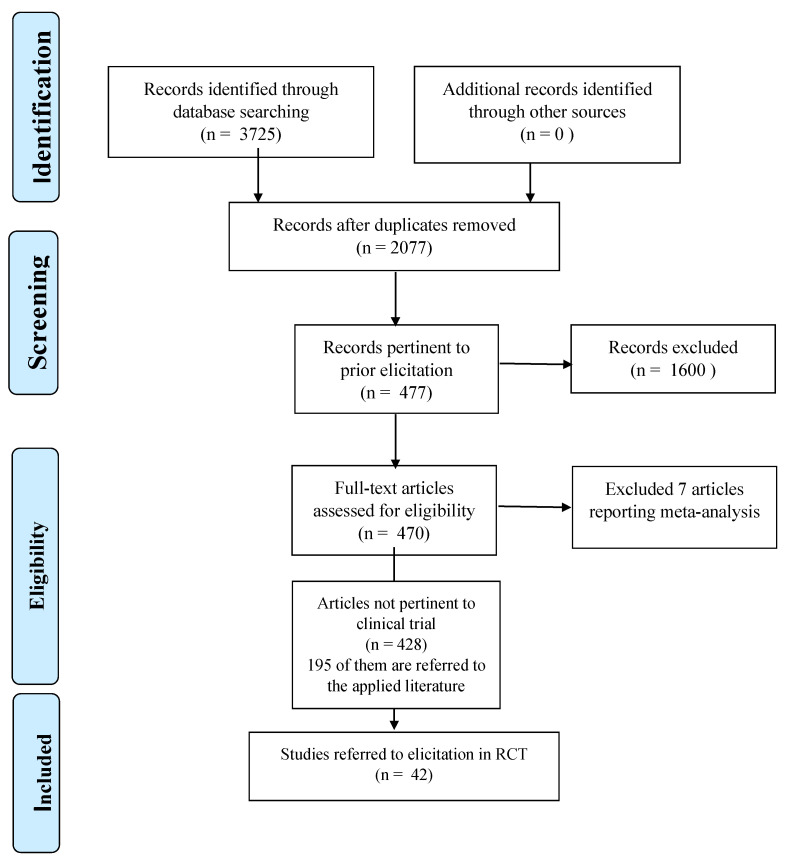
Prisma Flowchart.

A total of 3725 articles have been found performing the literature review. Among them, 470 articles are identified as pertinent to the Bayesian prior elicitation theme (Figure 1). Of these, 213 are published in Statistical Journals according to Journal Citation Reports^®^ classification [23].

**Figure 2:**
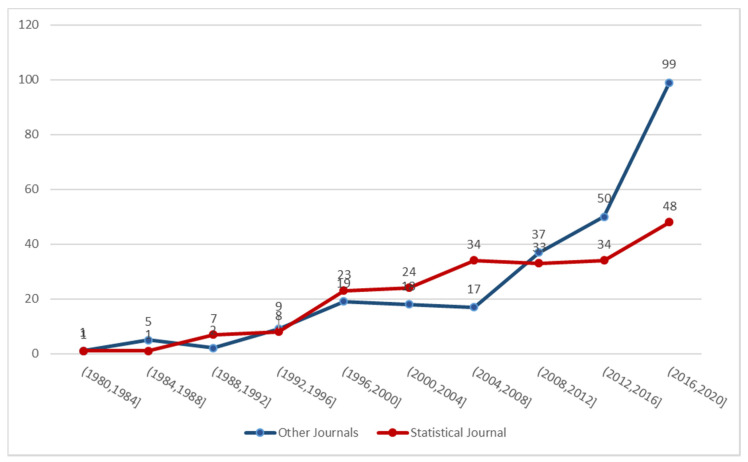
Articles pertinent to Prior Elicitation (n = 470) according to Journal type and year.

As per the temporal pattern of the prior elicitation literature, it is possible to observe that, until 2010, there is a greater number of publications in the statistical literature compared to other research areas; the pattern is reversed starting from 2009 to November 2020 (Figure 2).

Concerning the clinical trial research setting, it is possible to observe that 42 articles out of 470 deal with this research argument. Moreover, according to temporal trends, an increase in the number of publications concerning clinical trials is observed over time; 2 articles between 1992 and 2000, 10 in the period comprised between 2001 and 2008, 15 between 2009 and 2016, and 15 between 2017 and 2020.

### 3.2. State-of-the-Art Prior Elicitation in the Clinical Trial

Table 1 shows the characteristics of the 42 papers pertinent to clinical trial literature.

#### 3.2.1. Parametric Approaches

##### Continuous and Time to Event Outcomes

Considering continuous and time to event outcomes, Normal or Log-Normal priors are the preferred distributions for the elicitation procedure in 22 research articles. 

The Normal prior distribution is used in several fields of application:

- Survival endpoints

The Gaussian distribution is a used solution to define priors on hyper-parameters for a survival function assuming a Weibull time to event shape relation [37]. The author proposed Bayesian methods for right-censored survival data for populations with a surviving (cure) fraction. A real dataset from a melanoma clinical trial has been considered for the application. The normal random variable has been considered to parametrise the Weibull scale hyperparameter in an uninformative manner with high variance [37]. In fact, according to the Bayes and Laplace postulate, the absence of information concerning the treatment effect may be translated into equal prior probabilities for a discrete event and a flat prior (high variance) for the continuous endpoints [71].

In several cases, log-hazard ratios are also modelled as a normal distribution. Histograms representing the prior beliefs of each investigator were constructed and interpolating the Log Hazard ratio with a Gaussian distribution [49,62]. This approach has been employed also in cancer survival studies by performing a weighted averaging pooling of expert opinions [42]. 

The poling of the expert opinion may be performed by calculating the average of the height of the prior distributions for each parameter value (average pooling) or computing a geometric mean of the original densities (logarithmic pooling). Both techniques allow for different weights to be given to each opinion depending on the clinician’s experience in the area under study [72].

- Models hyperparameters

The normal approximation of experts’ opinion is also implemented to model parameters of Bayesian logistic regression. The method models the response as a Bernoulli random variable assuming the regression coefficients as a mixture of three normal distributions reflecting increase, decrease, and no substantive change in the response [51]. 

Moreover, the Gaussian priors have been considered also to define the hyperparameters for an adjusted hierarchical model for the miscounting count predictions of the Poisson and negative binomial models. The parametrisation has been proposed and applied to a large randomised controlled trial on Chronic Obstructive Pulmonary Disease [50]. The author derived the prior parameters from the historical information by using an adaptive prior weighting approach, accounting for a potential prior-data conflict. The idea is to discounting the prior information whatever the prior-data conflict exists [73].

- Study design

The Normal distribution has been also proposed in multi-stage trials to elicitate a prior for the treatment effect estimation and then calculating the probability that the trial will produce a favourable outcome; the decision to proceed with a larger trial has been translated in a prior probability distribution incorporating the information provided by a smaller trial [30]. The treatment effect has been elicitated via univariate quartile elicitation method: the Gaussian prior parameters have been derived fitting a probability distribution on the expert quantiles via least-squares procedure [13]. 

The normal prior elicitation is also considered for the Bayesian clinical trial design. Recent research evaluates the prior impact on the observed data model by introducing the effective current sample size (ECSS) prior approach. Special emphasis is put on the robust mixture, power, and commensurate priors defined on a normal and beta parametrisation [69].

The Gaussian random variable is considered in literature also to perform a prior elicitation for a survival function in the context of a Bayesian clinical trial planning [55]. The author proposes an assurance method, which is an alternative to a power calculation analysis based on the probability of a successful trial outcome computation via Elicitation of a prior probability distribution about the study treatment effect. The prior distribution for the difference in the time point-specific survival rate between treatment and control arm has been elicitated via univariate quantile elicitation method [13] by using a truncated Gaussian prior ensuring the support of the time-specific survival rate would be comprised between 0 and 1 [55].

- Treatment effect estimation

Another application of this prior parametrisation is used to elicitate the mean change score in a rare disease trial once the consensus among the experts has been reached [54]. The study endpoint under consideration was a mean change score measured 100 mm visual analog scale (VAS) assumed to follow a Gaussian distribution. The prior parameters were elicited by averaging individual quantile opinions among experts. 

In other cases, continuous outcomes, defined on log scales, are modelled eliciting experts’ opinions with normal distributions [59]. Individual responses achieved from the graphical elicitation method were summed and normalised to obtain a unique prior distribution which is the mean of the single expert normal priors [59].

Additionally, cost data, typically highly skewed, are elicited using Log-Normal transformation [60]. The research showed that the use of genuine prior information can provide more realistic conclusions in particular for cost-effectiveness analyses of trial data where sample sizes are relatively small. A genuine prior is represented by an informative distribution which assumes a higher probability to some values than to others within parametric space [60].

- Multivariate distributions and mixture of priors

In several cases, the overall prior distribution is developed by a weighted mixture of the single expert priors [63]. The mixture of expert’s Log-Normal priors has been used also to elicitate a prior distribution for the sources of bias affecting the final trial estimate which may be reported in a meta-analysis. The elicited opinions are used to develop prior distributions representing the biases in each study useful to perform a bias-adjusted meta-analysis [64]. 

A Bayesian method has been proposed and applied to a colon cancer trial where the expert information is used to perform a variable selection procedure [34]. A bivariate Normal random variable has been considered to parametrise the covariate effect, instead, a Beta variable is used to define the covariate weight in the feature selection procedure. The expert’s opinions have been pooled using a Bayesian Model Average approach [74].

In the CHARM clinical trial, the log-hazard of cardiovascular death has been modelled via Multivariate Normal distribution [68]. The analysis has been performed on a specific group of patients; the group-specific treatment effects have been estimated by using a Bayesian approach with informative Multi-Normal priors obtained eliciting expert’s opinions, interpolating the single opinion histogram with a normal random variable, and averaging across opinions.

The bivariate normal parametrisation has been also considered, in a two-stage phase I–II clinical trial design to optimise dose–schedule regimes where a flexible Bayesian hierarchical model has been used to account for the relation among patients subgroups and treatment regimens [48].

Other parametric distributions are considered in the literature for the continuous outcomes, for example, surgical learning curve parameters (first procedure and plateau level) are obtained averaging different experts’ opinion, using a power–law function [39].

The inverse gamma distributions served, instead, to model the accrual rate monitoring in a clinical trial. The posterior predictive accrual distribution has been obtained combining prior information on the accrual rate, provided by experts or historical data, with the information known up to the monitoring time point [40].

In another case, Laplace’s and Jeffreys’s priors are elicited to estimate a competing risks model with covariates [75]. Laplace’s prior has been considered for nonidentifiable model parameters, instead, Jeffreys’s prior has been considered for identifiable parameters [75].

Laplace’s distribution is a continuous probability distribution also note as a double exponential because its density can be seen as the association of two densities of exponential laws. Laplace’s law can also be obtained from the difference of two independent exponential variables with the same parameter [76]. This distribution has been used extensively as a sparsity-inducing mechanism to perform feature selection simultaneously within classification or regression. The mechanism is implemented in the LASSO regression. This prior places stronger confidence on zero regression coefficient than does a normal prior centred on zero [77].

The Jeffreys prior instead is a non-informative prior distribution. In agreement with the Jeffreys rule, a prior distribution is uninformative if its density function is proportional to the square root of the determinant of the Fisher information matrix [71,78].

##### Categorical Outcomes

Generally, prior probability distributions for binary outcomes are elicited in terms of Beta priors [31,45,46,56,58]. Aupiais and colleagues, for example, proposed a non-inferiority approach, in a Bayesian framework, for sequential monitoring of rare dichotomous safety events, incorporating experts’ opinions to define the margin. The acceptable difference between adverse event rates across arms, according to the expert opinions, was modelled using a mixture of beta distributions [31].

- SHELF elicitation procedure

The SHELF elicitation procedure is a widely used approach to elicitate Beta event rate in a clinical trial [12] and is the most commonly used software for elicitation (Table 1). Jansen et al. [45], for example, elicited the prior distribution for the 24-h trauma mortality in patients with haemorrhagic complications combining beta distributions using the SHELF elicitation procedure. The single expert distributions were elicited using the roulette method than a linear poling of the distributions has been performed [45].

In the roulette method, the expert provides probabilities of the treatment effect lying in a particular “bin” by allocating “gaming chips” to that bin. The method provides a graphical representation of the provided expert beliefs [14].

Another research underlines the feasibility of a SHELF elicitation procedure for the evaluation of drug safety or efficacy in a hypothetical early-stage trial. A beta prior has been considered for the elicitation of the expert’s opinions [46].

A sequential update of the experts’ opinion is also reported in veterinary trials by using a SHELF elicitation procedure on the beta event rate. This research has demonstrated the usefulness of probabilistic elicitation for evaluating the diversity and strength of experts’ beliefs concerning the efficacy of systemic antibiotics as dry cow therapy [43].

This software is often used for the computer-based elicitation procedure; the distributions are interactively elicited by showing to the experts the priors obtained through the software. Other elicitation procedures are based on (1) informal discussion (2) structured questionnaires (3) Structured interviewing with poling of opinions [72].

- Dose-response curves

In some cases, a normal distribution has been assumed on parameters characterising the dose-toxicity curve in Phase I clinical trial [32]. A phase I clinical trial is generally aimed to find a maximum tolerated dose, which is often a monotonically increasing dose-response curve following a logistic distribution. For example, the definition of a toxicity response may be based on the approach of eliciting a range for the probability of toxicity at the lowest dose level, and the value of the maximum tolerated dose. The prior for both parameters distribution may be considered as a uniform distribution over these ranges [57]. A non-parametric shape function, for a maximum, tolerated dose may also be reported. Another option, addressed in the literature, is the of the toxicity probability at each dose level considering a Beta prior distribution [38].

The Log-Normal and Normal prior parametrisation has been used also to develop generalised priors for different Bayesian Dose–Response parametric models [67].

A parametric distribution is also adopted in the literature for categorical endpoints by using the log transformation of odds ratios modelling binary data using elicited Normal priors [5]. Opinion on the relative efficacy of treatment was modelled as a normal distribution, the parameters of which were determined by asking experts questions concerning the distribution quantile.

#### 3.2.2. Non-Parametric Approaches

A total of eight articles [9,33,36,47,52,53,61,70] out of 42 treating expert elicitation in clinical trials consider non-parametric methods for the elicitation of the expert opinion. The principal non-parametric approaches applications are classified within:

- Histogram approach

Graphical visualisation of the expert opinion in histogram defined on parameters of a log-hazard function is a possible approach used to perform elicitation of the expert opinion. The method is flexible leading to define hazard regression coefficient with parametric distributions also allowing for non-parametric adjustments using more general copula combinations of marginal distribution [36]. Individual expert histograms representing the prior beliefs about the treatment effect are also used in other cases to derive non-parametric prior averaging individual expert opinion [9,61].

- Study design and power prior approach

The use of historical information to define the prior distribution in a non-parametric context is a method recently used in the literature [53]. Informative prior elicitation is typically a challenging task even in the presence of historical data (objective prior) [79]. Ibrahim and Chen [80] proposed the power prior approach to incorporate the historical data in the analysis of a current study. The method is based on the raising of the likelihood function of the historical data to a power parameter between 0 and 1 (power parameter). This parameter represents the proportion of the historical data incorporated in the prior. Diaconis and Ylvisaker [81] and Morris [13] studied conjugate priors for the exponential families by assuming a fixed power parameter. Ibrahim and Chen [80] considered the uncertainty component on power parameters.

The approach is widely used for the design and analysis of clinical trial data. The method is useful for handling problems related to a lack of exchangeability between the historical and current data, and the risk that prior information overwhelms the clinical trial data information [82].

In a sequential clinical trial, for example, a power prior approach is considered to weight the prior information together with the ESS (Effective Sample Size) approach is used to set the maximum desired amount of information to be shared from historical data at each step of the trial [52]. The ESS method leads to define the prior in terms of the number of hypothetical patients used to develop the prior. The procedure leads to quantify how is informative a prior distribution [83].

Recently, some efforts are evidenced in the literature to incorporate, in the study design phase, the alternative procedure to the prior definition. The method is tailored on a phase IIA trial and represents a Bayesian counterpart of a Simon two-stage design using historical data and semi-parametric prior’s elicitation methods [33].

- Dose findings in early phase trials

Non-parametric approaches are also considered to find a maximum tolerated dose in Phase I clinical trials using the Continual Reassessment Method design and proposing a suitable informative prior distribution on the relationship between outcome data and covariates [47,84]. In a dose-finding trial, non-parametric elicitation procedures are used eliciting expert quantiles opinion corresponding to the toxicity probability at each dose level [70].

#### 3.2.3. Field of Application

Phase II-III trial. The prior elicitation has been applied (16 studies) to the trials for an efficacy assessment within phase II or III trials (Table 1). The priors are defined for the drug efficacy assessment, especially in an informative setting (Table 1). However, in several cases, sensitivity analyses to the prior choices have been proposed, including both the results for the non-informative and informative analysis [31,37,61,63,68]. Concerning the prior distribution sensitivity analysis, the robust Bayesian approach has been proposed by Greenhouse and Wasserman and applied to the clinical trial data, especially to help the monitoring committee to decide whether or not early stopping a trial. The method investigates how the inferences might change as the prior varies over a class of distributions [85].

In other cases, different hypotheses are defined on the informative prior parameters [9,62]. Different levels of discounting are also considered on the historical information incorporated in the prior definition by using a power prior approach [56].

Early Phase I-II. Seven studies implemented the prior elicitation in early phases trial for the safety assessment (Table 1); the greater part of them (4) considered informative priors [38,47,48,52].

### 3.3. Topic Model Analysis

The analysis was performed on textual data of 470 articles. Two topics were selected for analysis because the maximum value of the Deveaud metric is 2.3 and has been reached in correspondence of two topics. Among the most frequent words (Figure S1, Supplementary Material), the redundant words (“result”, “assess”, “data”, “probabl”, “approach”, “propos”, “provid”, “base”, “knowledge”, “approach”, “develop”, “perform”, “also”, “present”) have been removed from the LDA computation algorithm.

The features pertinent to each topic are shown in Table 2. The most pertinent word on each topic, allow to characterise them by their structure of meaning.

1. The first one, here in after referred to as applied topic, is more related to the empirical application of the prior elicitation methods
2. The second topic, here in after referred to as theoretical topic, seems to be related to the theoretical implications of the prior elicitation procedure

Table 1 shows that 28 papers are manually classified as applied works (applied topic), and 14 papers concern a theoretical topic. The articles reporting both theoretical and practical applications have been classified as applied topic papers. The overall accuracy computed on manually screened 42 trial articles is equal to 83% (7 articles have been misclassified by the LDA algorithm).

**Figure 3:**
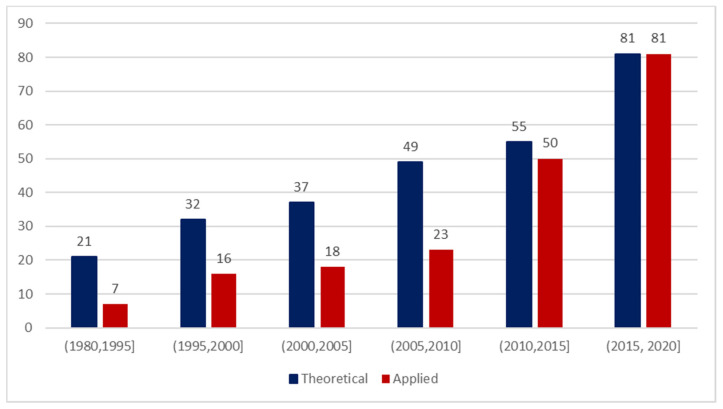
Classification of prior elicitation pertinent articles according to LDA topics and publication years.

Observing the predictions of the LDA algorithm according to publication year (Figure 3) it is possible to observe that the prior elicitation procedure is prevalently addressed in Theoretical topic literature until 2010. The pattern is reversed in recent years evidencing an increasing interest on prior elicitations methods also in the generally applied research literature. The number of published papers concerning the prior elicitation increases both in a theoretical and applied framework. This growth continues in parallel with the increase of interest of the scientific literature for the Bayesian approach in general. The pattern of publication of papers containing the word “Bayes” on Pubmed (Figure S2, Supplementary Material), we observe a relevant growth starting from the first half of 2000.

Moreover, comparing LDA results about trial articles with the overall pertinent literature on prior elicitation, it is possible to observe a greater proportion of applied papers in trial pertinent literature, and evidence that a consistent part of theoretical literature is allocated in not pertinent articles (Table 3).

## 4. Discussion

Study findings indicate that starting from 2010, it is also possible to observe a spreading of the prior elicitation techniques in research fields different from the theoretical statistics. This aspect may be related to the recent increase in the popularity of the Bayesian methods in a general setting and the clinical trial research [2]. In recent years, Bayesian methods have increasingly being used in the design, monitoring, and analysis of clinical trials due to their flexibility [86].

The increase in popularity of Bayesian methods in a clinical trial involves a need for statisticians to define tools useful for the definition of robust and defensible informative prior distributions [87].

Empirical data may be used to define such priors (objective prior) whenever possible. However, in some cases, the limitations in data availability may preclude the construction of a data-based prior. In this situation, an expert elicitation procedure may be a solution used to define prior distributions [87].

In clinical trial publications, parametric distributions are mostly employed in applied settings. Semi-parametric or non-parametric priors are poorly used within this field, confining them mainly to the theoretical field. This aspect concerns especially less diffused approaches involving non-parametric methods for prior elicitation method. The reason behind the limited application of the non-parametric methods is surely related to the computational effort associated with the definition of a prior distribution which is more flexible and adaptable to the expert opinion but, in several cases, leads to obtaining posterior distributions difficult to be expressed in the closed-form [88].

It is important to consider that, in some research contexts, the translation of the experts’ opinions into a pre-specified family distribution may be considered a limitation because many different distributions may be more suitable to the experts’ opinions generally expressed in quantiles [14].

In recent years, not only parametric but also non-parametric methods to the elicitation of expert opinion are treated especially in the theoretical literature.

However, in clinical trial research, the conventional parametric methods are the more adopted procedures to the elicitation of expert opinion, leaving non-parametric methods predominantly in a statistical field.

Given the potential of prior elicitation to a better decision on making, more efforts are needed to ensure diffusion of the prior elicitation facilities, not only in theoretical statistical research but also in applied clinical trial settings, both at the design and analysis stage.

## 5. Conclusions

The prior elicitation methods are recently appealing not only to statistical literature but also in other research settings. It is possible to observe that the methods are increasingly used in general literature and clinical trial research.

However, in this framework, conventional parametric methods are more popular in clinical trial research. The non-parametric approaches are, in several cases, treated specially in the theoretical literature which is mainly focused on a statistical argumentation.

## Figures and Tables

**Table 1:** Articles treating prior elicitation in clinical trial classified according to publication year, first author, title, main approach, the prior distribution, trial phase, prior information, software used for elicitation, manual classification.

Publication Year	Author	Title	Approach	Prior Distribution	Trial Phase	Prior Information	Software	Manual Classification
2020	(Alhussain et al., 2020) [30]	Assurance for clinical trial design with normally distributed outcomes: Eliciting uncertainty about variances	Parametric	Normal	Study design	Sensitivity with different levels of prior variability	SHELF	Applied
2019	(Aupiais et al., 2019) [31]	A Bayesian non-inferiority approach using experts’ margin elicitation-application to the monitoring of safety events	Parametric	Beta	Phase III monitoring	Non-informative Beta and informative with parameters defined by expert opinions	Betareg R	Applied
2004	(Bekele & Thall, 2004) [32]	Dose-finding based on multiple toxicities in a soft tissue sarcoma trial	Parametric	Multinormal	Phase I	Sensitivity analysis by randomly perturbing the elicited toxicity weight vector	None	Applied
2019	(Berchialla et al., 2019) [33]	Bayesian sample size determination for phase IIA clinical trials using historical data and semi-parametric prior’s Elicitation	Semiparametric	B-Spline	Study design	Uninformative and best fit to the expert opinions	None	Theoretical
2019	(Boulet et al., 2019) [34]	Bayesian variable selection based on clinical relevance weights in small sample studies-Application to colon cancer	Parametric	Mixture of normal	Not applicable	Informative	None	Applied
2017	(Browne et al., 2017) [35]	A Bayesian Analysis of a Randomised Clinical Trial Comparing Antimetabolite Therapies for Non-Infectious Uveitis	Parametric	Normal	Phase II–III	Informative	Matematica and R	Applied
1993	(Chaloner et al., 1993) [36]	Graphical Elicitation of a prior distribution for a clinical trial	Non-Parametric	Non-parametric adjustments via copula combinations of the marginal distribution	Phase II–III	Informative	XLISP-STAT software	Theoretical
1999	(Chen et al., 1999) [37]	A new Bayesian model for survival data with a surviving fraction	Parametric	Gamma and Normal	Phase III	Non-informative and Informative prior	None	Theoretical
2002	(Cheung, 2002) [38]	On the use of nonparametric curves in phase I trials with low toxicity tolerance	Parametric	Beta	Phase I	Informative	None	Theoretical
2012	(Cook et al., 2012) [39]	A questionnaire elicitation of surgeons’ belief about learning within a surgical trial	Parametric	Learning curve	Phase II	Informative	None	Applied
2008	(Gajewski, Simon, & Carlson, 2008) [40]	Predicting accrual in clinical trials with Bayesian posterior predictive distributions	Parametric	Inverse Gamma	Not applicable	Non-Informative or informative	Accrual R package	Theoretical
2015	(Hampson et al., 2015) [41]	Elicitation of Expert Prior Opinion: Application to the MYPAN Trial in Childhood Polyarteritis Nodosa	Parametric	Beta and Normal	Phase III	Informative	Shiny app created by authors	Applied
2009	(Hiance et al., 2009) [42]	A practical approach for eliciting expert prior beliefs about cancer survival in phase III randomised trial	Parametric	Normal	Phase III	Informative	None	Applied
2011	(Higgins et al., 2011) [43]	A Bayesian approach demonstrating that the incorporation of practitioners’ clinical beliefs into research design is crucial for effective knowledge transfer	Parametric	Beta	Not applicable	Informative	SHELF	Applied
2012	(Higgins, Dryden, & Green, 2012) [44]	A Bayesian elicitation of veterinary beliefs regarding systemic dry cow therapy: Variation and importance for the clinical trial design	Parametric	Beta	Not applied	Informative	SHELF	Applied
2020	(Jansen et al., 2011) [45]	Elicitation of prior probability distributions for a proposed Bayesian randomised clinical trial of whole blood for trauma resuscitation	Parametric	Beta	Phase III	Informative	SHELF	Applied
2011	(Johnson et al., 2011) [9]	Effect of warfarin on survival in scleroderma-associated pulmonary arterial hypertension (SSc-PAH) and idiopathic PAH. Belief elicitation for Bayesian priors	Non-parametric	Density histogram	Phase III	Sensitivity analysis to different Informative priors	None	Applied
2013	(Kinnersley, N.; Day, S., 2013) [46]	A structured approach to the Elicitation of expert beliefs for a Bayesian-designed clinical trial: A case study	Parametric	Beta	Phase II	Informative	SHELF	Applied
2001	(Legedza & Ibrahim, 2001) [47]	Heterogeneity in phase I clinical trials: prior Elicitation and computation using the continual reassessment method	Non-parametric	Ibrahim Prior (Ibrahim et al., 1998)	Phase I	Informative	S plus	Theoretical
2020	(Lin et al., 2020) [48]	An adaptive trial design to optimise dose-schedule regimes with delayed outcomes	Parametric	Bivariate Normal	Phase I–II clinical trial design	Informative	None	Applied
2013	(Moatti et al., 2013) [49]	Modeling of experts’ divergent prior beliefs for a sequential phase III clinical trial	Parametric	Mixture of normal	Phase III	Informative	Mixdist package R	Theoretical
2018	(Muff et al., 2011) [50]	Bias away from the null due to miscounted outcomes? A case study on the TORCH trial	Parametric	Normal Log-Normal	Phase III	Informative	None	Applied
2009	(O’Leary et al., 2009) [51]	Comparison of three expert elicitation methods for logistic regression on predicting the presence of the threatened brush-tailed rock-wall by Petrogale penicillata	Parametric	Normal and Multinormal	Not applicable	Informative and Non-Informative prior	GIS, a map-based software developed by authors	Theoretical
2020	(Ollier et al., 2011) [52]	An adaptive power prior for sequential clinical trials-Application to bridging studies	Non-parametric	Ibrahim power Prior (Ibrahim et al., 1998)	Phase I	Informative	None	Theoretical
2019	(Psioda et al., 2011) [53]	Bayesian clinical trial design using historical data that inform the treatment effect	Non-parametric	Ibrahim adaptive power Prior (Ibrahim et al., 1998)	Study design	Informative	None	Theoretical
2019	(Ramanan et al., 2011) [54]	Defining consensus opinion to develop randomised controlled trials in rare diseases using Bayesian design: An example of a proposed trial of adalimumab versus pamidronate for children with CNO/CRMO	Parametric	Normal	Phase II	Informative	Shiny web app proposed by authors	Applied
2014	(Ren & Oakley, 2014) [55]	Assurance calculations for planning clinical trials with time-to-event outcomes	Parametric	Log-Normal	Study design	Informative	Software implemented by the authors	Theoretical
2011	(Rietbergen et.al., 2011) [56]	Incorporation of historical data in the analysis of randomised therapeutic trials	Parametric	Beta	Phase II–III	Informative with power prior	None	Theoretical
2005	(Rosenberger et al., 2005) [57]	Development of interactive software for Bayesian optimal phase 1 clinical trial design	Parametric	Uniform	Phase I	Uninformative by default	IDose software	Applied
2005	(Rovers et al., 2005) [58]	Bayes’ theorem: A negative example of an RCT on grommets in children with glue ear	Parametric	Beta	Not applicable	Informative	None	Applied
2012	(See et al., 2012) [59]	Prior Elicitation and Bayesian Analysis of the Steroids for Corneal Ulcers Trial	Parametric	Mixture of normal	Phase 3	Informative	Mathematica	Applied
2002	(Stevens & O’Hagan, 2002) [60]	Incorporation of genuine prior information in cost-effectiveness analysis of clinical trial data	Parametric	Log-Normal	Not applicable	Informative and Non-Informative prior	None	Applied
2013	(Sun et al., 2013) [61]	Expert Prior Elicitation and Bayesian Analysis of the Mycotic Ulcer Treatment Trial I	Non-Parametric	Density histogram	Phase III	Informative and Non-Informative prior	Mathematica	Applied
2003	(Tan et al., 2003) [62]	Elicitation of prior distributions for a phase III randomised controlled trial of adjuvant therapy with surgery for hepatocellular carcinoma	Parametric	Normal	Phase III	Sensitivity to informative priors	None	Applied
2017	(Thall et al., 2017) [63]	Bayesian treatment comparison using parametric mixture priors computed from elicited histograms	Parametric	Mixture of Normal	Phase II–III	Informative and Non-Informative prior	None	Theoretical
2009	(Turner et al., 2009) [64]	Bias modeling in evidence synthesis	Parametric	Log-Normal	Not applicable	Informative	None	Applied
2017	(Veen, Stoel, Zondervan-Zwijnenburg, & van de Schoot, 2017) [65]	Proposal for a Five-Step Method to Elicit Expert Judgment	Parametric	Normal	Not applicable	Informative	MATCH	Applied
2003	(Wang & Ghosh, 2003) [66]	Bayesian analysis of bivariate competing risks models with covariates	Parametric	Laplace and Jeffreys Prior	Not applicable	Informative and Non-Informative prior	None	Theoretical
2019	(Wheeler et al., 2011) [67]	Quantal Risk Assessment Database: A Database for Exploring Patterns in Quantal Dose-Response Data in Risk Assessment and its Application to Develop Priors for Bayesian Dose-Response Analysis	Parametric	Beta and Log-Normal	Not applicable	Informative and Non-Informative prior	None	Applied
2005	(White et al., 2005) [68]	Eliciting and using expert opinions about the influence of patient characteristics on treatment effects: a Bayesian analysis of the CHARM trials	Parametric	Normal	Phase III	Informative and Non-Informative prior	None	Applied
2020	(Wiesenfarth et al., 2005) [69]	Quantification of prior impact in terms of effective current sample size	Parametric	Normal and Beta prior	Study design	Informative and Non-Informative prior	None	Applied
2011	(Zohar et al., 2011) [70]	Planning a Bayesian early-phase phase I/II study for human vaccines in HER2 carcinomas	Non-parametric	Non-parametric expert quantiles distribution	Phase I–II	Informative and Non-Informative prior	None	Applied

**Table 2:** Pertinent words according to each LDA topic. In bold are represented the most important words.

	Applied	Theoretical
**1**	**study**	**model**
**2**	**effect**	**distribut**
**3**	**estim**	**inform**
**4**	**opinion**	**paramet**
**5**	**uncertainti**	**posterior**
**6**	test	sampl
**7**	process	function
**8**	risk	paper
**9**	practic	statist
**10**	case	predict

**Table 3:** Classification of articles according to LDA topics and pertinence to the clinical trial literature.

	Applied	Theoretical	Total
Pertinent to clinical trials	16% (31)	4% (11)	42
Not pertinent to clinical trials	84% (164)	96% (264)	428
Total	195	275	470

## Data Availability

Data sharing not applicable.

## Supplementary Materials

The following are available online at https://www.mdpi.com/1660-4601/18/4/1833/s1, Figure S1: Most frequent word within the Document Term Matrix, Figure S2: PubMed published articles containing the word “Bayes” according to the publication year.
