# Supplementary material for: Prior Elicitation for Use in Clinical Trial Design and Analysis: A Literature Review
Source: Int J Environ Res Public Health. 2021 Feb 13;18(4):1833. doi: 10.3390/ijerph18041833 (PMC7917693; doi:10.3390/ijerph18041833)

Supplementary Material

Figure S1 Most frequent word within the Document Term Matrix

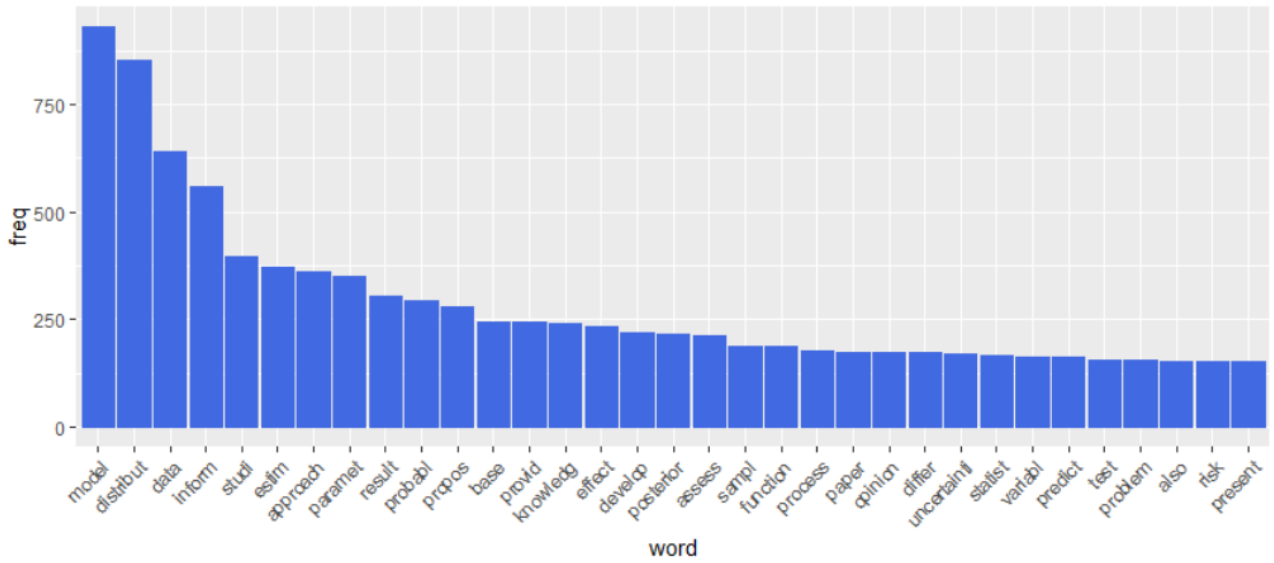

Figure S2 Pubmed published articles containing the word “Bayes” according to the publication year.

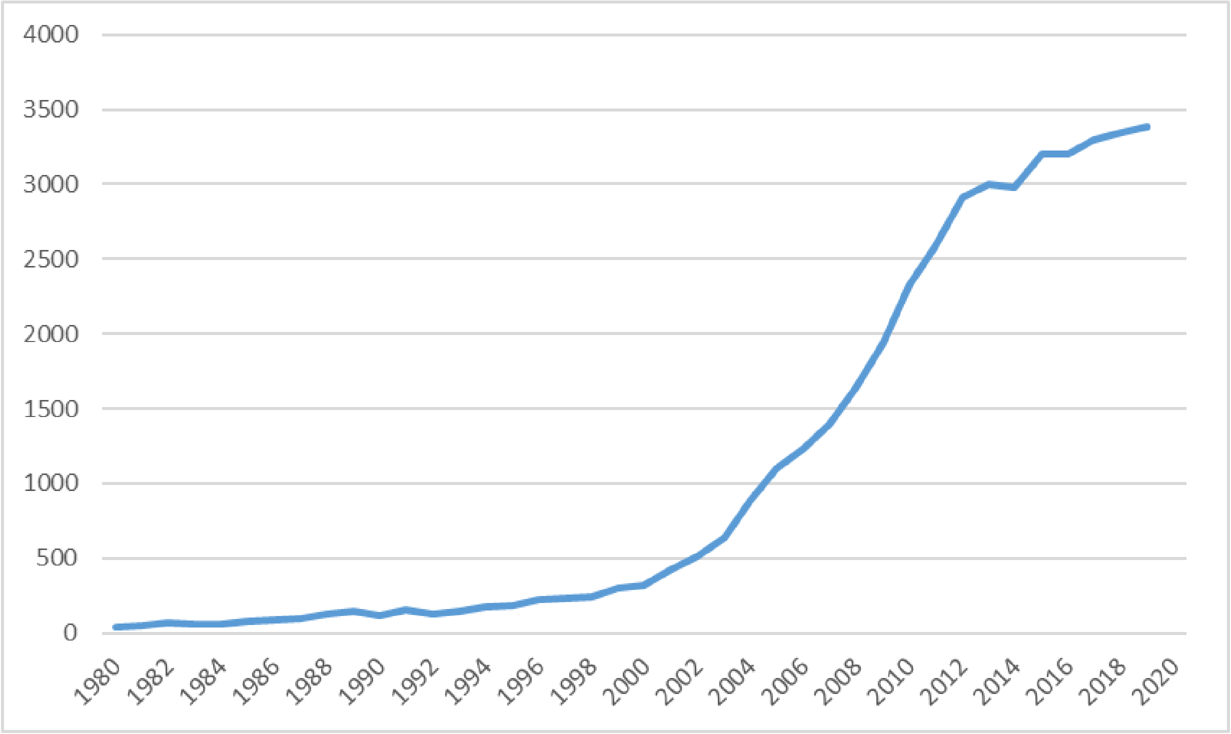

Supplement: Supplementary file 1 [file ijerph-18-01833-s001.pdf]
